# Metabolics-Based Study on the Therapeutic Mechanism Behind the Effect of Shenhuang Plaster Applied to the Shenque Acupoint on Gastrointestinal Motility in POI Mice

**DOI:** 10.3390/metabo15010065

**Published:** 2025-01-20

**Authors:** Yanan Shi, Chenglei Wu, Ting Liu, Rongyun Wang, Bin Ding, Qiuhua Sun

**Affiliations:** 1 The College of Nursing, Zhejiang Chinese Medical University, Hangzhou 310053, China; shiyanan@zcmu.edu.cn (Y.S.); 202411114111045@zcmu.edu.cn (C.W.); 20231035@zcmu.edu.cn (T.L.); 20211017@zcmu.edu.cn (R.W.); 2 College of Life Science, Zhejiang Chinese Medical University, Hangzhou 310053, China

**Keywords:** Shenhuang Plaster, Shenque acupoint, gastrointestinal motility, postoperative ileus, metabolomics

## Abstract

Background: Postoperative ileus (POI) is a common postoperative clinical complication that significantly affects postoperative rehabilitation and quality of life in patients and can even produce secondary complications, leading to serious consequences. External treatment using Shenhuang Plaster (SHP) (Shenque acupoint administration) has definite effects and unique advantages in the prevention and treatment of POI, but its mechanism is not completely clear. In this study, we investigated the therapeutic mechanism behind the effect of Shenhuang Plaster applied to the Shenque acupoint on gastrointestinal motility in POI mice based on metabolomics. Materials and Methods: C57BL/6 mice were divided into three groups: blank control (Ctrl), model (POI), and intervention (POI + SHP) groups. SHP treatment was started 3 days before modeling. We employed several behavioral tests and gastrointestinal transit function measurements and performed qRT-PCR analysis, 16S rRNA gene sequencing, and metabolomics analysis on serum metabolites. Results: We found that SHP could reduce the mRNA expression of inflammatory mediators in the smooth muscle tissue of the small intestine, regulate the structure and function of the intestinal microbiota, and modulate serum phenylalanine, carnitine, and glutamic acid levels. Conclusions: POI mice had obvious intestinal flora disorders and metabolic disorders of amino acids and their derivatives, and there was a significant correlation between differential flora and differential metabolites. SHP could effectively regulate the concentration of intestinal flora and serum metabolites and the metabolic pathway related to amino acids in vivo and, ultimately, achieve a therapeutic purpose in POI. In this study, it was found, for the first time, that applying SHP to the Shenque acupoint could effectively regulate the serum metabolites of phenylalanine, carnitine, and glutamate, and improve postoperative intestinal motile disturbance through association with the intestinal flora.

## 1. Introduction

Postoperative ileus (POI) is a common complication from surgery, characterized by a persistently high incidence rate. POI can lead to restricted gastrointestinal motility and delayed gastric emptying, resulting in the accumulation of many bacteria in the gastrointestinal tract, the accelerated absorption of toxins, the activation of intestinal immune cells, and increased release of inflammatory factors [1]. Additionally, POI prolongs hospital stays, increases hospitalization costs, and severely impacts patients’ postoperative recovery [2]. Therefore, the exploration of effective interventions for POI has garnered significant academic attention.

At present, intervention measures in Western medicine in the treatment of POI mainly include early postoperative fluid rehydration to correct electrolyte disturbance, enteral feeding to enhance resistance and improve nutritional status, and the use of gastric motility drugs such as dopamine receptor antagonists. However, there is still a lack of intervention and treatment methods with obvious effects in clinical practice, meaning that the incidence of POI is still high, which not only increases pain in patients and affects postoperative rehabilitation and the possibility of secondary complications, but also increases the medical burden on society and family.

Based on Traditional Chinese Medicine (TCM) theory, the use of Chinese herbal medicines and suitable TCM techniques in POI intervention has received extensive clinical validation and shows promising prospects for broader application. TCM posits that the pathogenesis of gastrointestinal motility disorders mainly involves weakness in the spleen and stomach and disordered qi activity. “Qi” is a central concept in traditional Chinese medicine theory. It is one of the basic substances that constitute the human body and maintain its life activities. It has the role of promoting the movement and metabolism of substances such as blood and body fluids, while also regulating various physiological functions in the human body. Treatment typically focuses on tonifying qi by purgation and regulating the qi activity of the spleen and stomach through methods such as the oral administration of Chinese herbs to promote gastrointestinal motility, acupuncture to enhance physical functions and alleviate gastrointestinal motility disorders to promote gastric emptying, Chinese medicine enemas for local absorption to protect the intestinal mucosal barrier, and acupoint application to restore gastrointestinal function [3,4,5]. Techniques such as acupuncture, Chinese medicine enemas, and acupoint application are effective, easy to perform, low-cost, and have few side effects, demonstrating unique advantages in interventions in gastrointestinal motility disorders.

Our previous research suggests that using TCM external treatment, specifically the application of a Chinese medicine ointment to the Shenque acupoint (navel therapy), can promote the recovery of gastrointestinal motility in POI, possibly due to the combined effects of the herbal medicine and the acupoint. However, the exact mechanism of action is not fully understood and requires further exploration. Based on the clear efficacy and unique advantages of TCM external treatment (Shenque acupoint medication) in preventing and treating POI, in this project, building on previous research, we constructed a POI mouse model and applied Shenhuang Formula as a Chinese medicine plaster to the abdomen (Shenque acupoint) of mice. We monitored and analyzed the effects of Shenhuang Plaster (SHP) on gastrointestinal transit function, the expression of inflammatory factors in intestinal tissues, the gut microbiota, and serum metabolites in POI mice to further investigate the mechanisms of SHP in POI mice.

## 2. Materials and Methods

### 2.1. Experimental Animals

Thirty SPF-grade C57BL/6 male mice, aged 6–8 weeks, weighing 19–22 g, were purchased from Shanghai Sippr-BK Laboratory Animal Co., Ltd. (Shanghai, China). The production license number for the experimental animals was SCXK (Hu) 2021–0006. After purchase, the mice were housed in a barrier environment of the school’s experimental animal center, with the animal use license number SYXK (Zhe) 2021–0012 (ethical approval number: IACUC-202312-12). The housing conditions were as follows: room temperature, 22–24 °C; relative humidity, 40–60%; light/dark cycle of an 08:00–20:00 light period and a 20:00–08:00 dark period; five mice per cage with sufficient food and water provided ad libitum.

### 2.2. Experimental Reagents

The experimental reagents and manufacturers used in this experiment were shown in the Table 1.

### 2.3. Experimental Apparatus

The experimental apparatus and manufacturers used in this experiment were shown in the Table 2.

### 2.4. Preparation of Shenhuang Plaster

The preparation of SHP adhered to the formulation, preparation process, and methods from our previous research on Shenhuang preparations. Through one-way and orthogonal tests, we optimized the extraction and purification process of the Shenhuang Formula, using the transfer rate and purity of quality markers as the main indicators. Final quality control was based on appearance properties, initial viscosity, viscosity retention, and the degree of release of quality markers. The preparation was completed in the Chinese Medicine Preparation Room of Zhejiang Chinese Medical University and used for animal experiments. The production steps were as follows: (1) ginsenosides were extracted from Renshen (ginseng). (2) Total anthraquinones were extracted from raw Da Huang (raw rhubarb). (3) A mixture of 300 g of Danshen (salvia miltiorrhiza), 200 g of Zhishi (citrus aurantium), 250 g of Houpo (magnoliae officinalis), 125 g of Wuzhuyu (euodia ruticarpa), and 75 g of Dingxiang (cloves) was prepared and refluxed twice with 8 times the amount of 80% ethanol, for 1 h each time. The ethanol was recovered to achieve an alcohol-free taste and freeze-dried. (4) An amount of 50 g of Dingxiang (clove) was crushed into coarse particles, added to 10 times the amount of water, soaked for 12 h, and extracted by steam distillation for 8 h to collect the aromatic water. The powders from steps (1), (2), and (3) above were uniformly mixed, and the aromatic water extracted from (4) was added as an osmotic agent. After mixing well, it was coated on the blank Babu paste backing to make the SHP Babu agent and stored in the refrigerator at 4 °C for later use. The extraction of the ingredients and the preparation of the SHP were carried out by the Traditional Chinese Medicine Formulation Laboratory at our university, and UPLC-MS was applied to identify the main effective components of SHP [6].

### 2.5. POI Modeling and Intervention Protocol

To construct a POI model, C57BL/6 mice were adaptively fed for one week and then randomly divided into three groups—blank control (Ctrl), model (POI), and intervention (POI + SHP) groups—with 10 mice in each group. The modeling method followed the protocol described by Kalff JC et al.: after isoflurane inhalation anesthesia in mice, abdomens were shaved with a small shaving machine and disinfected, and a sterile gauze was applied. Under sterile conditions, a 2 cm long incision was made along the midline of the lower abdomen to expose the abdominal cavity. The small intestine was turned to the left side and placed on a saline-moistened cotton pad. A wet cotton swab was pressed and pushed along the long axis of the small intestine, from the beginning to the end, to simulate the common clinical small intestine exploration operation, with the force required to push the intestinal contents but not penetrate the cecum. After pressuring, the small intestine was returned to the abdominal cavity in order, a small amount of warm saline was dripped into the cavity, and the abdominal wall was closed using a double-layer continuous suture. The entire surgical procedure was performed within 15 min. During the surgery and for 30 min post-operation, the mice were placed under a heating lamp. Throughout the procedure, physiological saline was intermittently administered to the surface of the intestines to prevent ischemic necrosis. When the mice recovered autonomous movement and could move independently, they were returned to the cage and kept warm, and any changes were observed [7].

After the POI model was established, in the intervention group, we used a cotton swab to apply an appropriate amount of SHP to the abdomen of mice (Shenque acupoint, CV8) twice a day. Acupoint localization for mice followed the standards described in *Experimental Acupuncture Science* by Li Zhongren, selecting the Shenque acupoint (the center of the navel on the midline of the abdomen). The control and model groups received saline applications in the same manner. Due to the slow onset of external acupoint treatment, the application of SHP began three days before modeling and continued until seven days post-modeling, for a total of ten days. The mice in each group were weighed regularly every day and observed for their mental state, fur color luster, and dietary status.

### 2.6. Gastrointestinal Transit Function Measurement

After 24 h of fasting, mice were administered 200 μL of FITC-labeled dextran (70 kDa) at a concentration of 6.25 mg/mL by oral gavage. Thirty minutes later, the mice were anesthetized and euthanized. The entire gastrointestinal tract was then removed and divided into 15 segments: stomach, small intestine (10 equal segments), cecum, and colon (3 segments). Each segment was flushed with saline to collect the FITC-labeled dextran into centrifuge tubes. The samples were centrifuged at 12,000 rpm for 15 min, and the supernatant was transferred to a black 96-well plate under dark conditions. The absorbance was measured at 494 nm using a microplate reader, and the percentage and geometric mean of absorbance for each segment were calculated. Gastrointestinal transit function was expressed as the percentage distribution of FITC-labeled dextran absorbance in each segment.

### 2.7. Detection of Inflammatory Mediator mRNA Expression in Small-Intestinal Smooth Muscle by qRT-PCR

Total RNA was extracted from the intestinal wall tissue using the Trizol method. The extracted RNA was quantified using a Nanodrop spectrophotometer and reverse-transcribed into cDNA using SuperScript III (Invitrogen, Carlsbad, CA, USA). The expression of the inflammatory cytokines IL-1β, IL-6, T-bet, and TNF-α mRNA was analyzed using a Bio-Rad iQ5 Multicolor Real-Time PCR Detection System (BioRad, Hercules, CA, USA). The specific primers used for amplification are listed in Table 3. The qRT-PCR procedure was conducted according to the SYBR^®^ Green PCR Master Mix kit instructions. β-actin was used as the internal control gene. The relative expression levels of the target genes in each sample were calculated using the 2-DDCt method.

DNA was extracted from 200 mg of frozen fecal samples using a fecal DNA extraction kit. The extracted DNA was quantified by NanoDrop (Thermo Scientific, USA) and then detected and purified by 1.0% agarose gel electrophoresis. The V3-V4 region of the 16S rDNA gene was amplified using universal primers (341F 5′-CCTACGGGNGGCWGCAG-3′ and 805R 5′-GACTACHVGGGTATCTAATCC-3′) with Phusion Hot Start Flex 2× Master Mix [8]. Sequencing libraries were constructed, and the libraries were quantified and assessed for quality using Qbuit and Aglient 2100 analyzers. Sequencing was performed on the Illumina NovaSeq platform with insert sizes of 275–450 bp. Clean data were assembled using SOAP de novo (v2.04), and gene prediction was performed using MetaGeneMark to construct a non-redundant gene set. Species annotation was conducted using Mytaxa and relevant databases, and the species abundance tables at different taxonomic levels were obtained by combining the gene abundance table. Species abundance tables and functional abundance tables were generated for further significant PCoA and sample clustering analyses.

### 2.8. Metabolomics Analysis of Serum Metabolites

Metabolite extraction: Serum was centrifuged to obtain the supernatant. A 20 μL sample was mixed with 120 μL of 50% methanol, vortexed for good mixing and to extract the metabolites from the sample, and left at room temperature for 10 min. The extraction solution was left at −20 °C overnight to precipitate proteins. The mixture was centrifuged at 4000 r/min for 20 min, and the supernatant was transferred to a 96-well plate. An equal volume of 10 μL from each sample was combined to form QC samples. All metabolite samples were stored at −80 °C before analysis. The liquid chromatography system used for data acquisition was SCIEX’s ultra high-pressure liquid chromatography. Liquid chromatography section: The column temperature was set at 35 °C, and the flow rate was 0.4 mL/min. The mobile phase consisted of A—water (1% formic acid) and B—acetonitrile (1% formic acid). The gradient settings were 0–0.5 min, 5% B; 0.5–7 min, 5–100% B; 7–8 min, 100% B; 8–8.1 min, 100–5% B; and 8.1–10 min, 5% B. High-resolution mass spectrometry was used, with each sample being analyzed in both positive- and negative-ion modes. The ion source conditions were as follows: 30 PSI for curtain gas, 60 PSI for auxiliary and sheath gasses, a source temperature at 650 °C, and voltages of +5000 V for positive-ion and −4500 V for negative-ion modes. Data were collected in information-dependent acquisition mode. Bioinformatics analysis: Raw files were converted to mzXML files using Proteowizard’s MSConvert software (ProteoWizard 3.0.6150) and imported into XCMS software (version 3.9.3) for peak extraction and retention time correction. These data matrices were then imported into SIMCA-P 14.0 software for multivariate statistical analysis. Partial least squares discriminant analysis (PLS-DA) was used to summarize metabolic profile differences, and orthogonal projections to latent structures discriminant analysis (OPLS-DA) was conducted to validate the PLS-DA model and further maximize the differentiation between groups. The model’s suitability and predictability were explained by R2Y and Q2 values, respectively. Potential candidate metabolites were filtered based on the following criteria: ANOVA-adjusted *p*-value < 0.05, fold change (FC) > 1.33 or <0.77, VIP > 1, and correlation coefficient |*r*| > 0.55.

### 2.9. Statistical Analysis

For the statistical analysis in this study, categorical data were expressed as rates or composition ratios, while continuous data were presented as X ± S. The analysis was performed using IBM SPSS 22.0 software. Pairwise comparisons between groups were conducted using the LSD-t test. For multiple-group comparisons, ANOVA was used when variances were homogeneous, and the Kruskal–Wallis H test was applied when variances were not homogeneous. All tests were two-tailed, and a *p*-value of less than 0.05 was considered statistically significant.

Omics data: We performed 16S sequencing data analysis using data exported from the Illumina NovaSeq 6000 sequencing platform and analyzed with RStudio software (R-3.4.4) and the online analysis tool LEfSe for gut microbiome-related data. Metabolomics data were obtained from the UPLC-TripleTOF 5600 platform, preprocessed with R software (v3.5.2), normalized using Excel 2010, and subjected to multivariate statistical analysis with SIMCA-P 14.0. Correlation analysis was conducted using Spearman’s correlation in R.

## 3. Results

### 3.1. General Behavioral Indicators of Mice in Each Group

During the experiment, the mental state, fur luster, and feeding status of the mice were closely observed. The mice in the Ctrl group maintained a good mental state, were active, had smooth and shiny fur, and exhibited normal feeding behavior throughout the experiment. In contrast, the POI group mice had dull, lusterless fur, were lethargic, huddled in corners, and hardly ate. After intervention with Shenhuang Plaster (SHP), the POI + SHP group mice showed improvements in their mental state, activity level, fur luster, and food intake compared to the POI group. Additionally, the body weight of the mice was monitored, with measurements taken every other day. Table 4 presents the weight changes in the mice in the three groups at different stages. The initial weight was the weight before modeling, and the final weight was the weight at the end of the experiment. The average weight gain for each group was calculated. The results showed that the weight gain in the POI group was significantly lower than that in the Ctrl group (*p* < 0.001), and this difference was statistically significant. Conversely, the weight gain in the POI + SHP group was significantly higher than that in the POI group (*p* < 0.05), and this difference was also statistically significant, indicating that SHP effectively restored food intake in the mice.

### 3.2. Changes in Gastrointestinal Distribution of Fluorescent Dextran in Mice in Each Group

**Figure 1:**
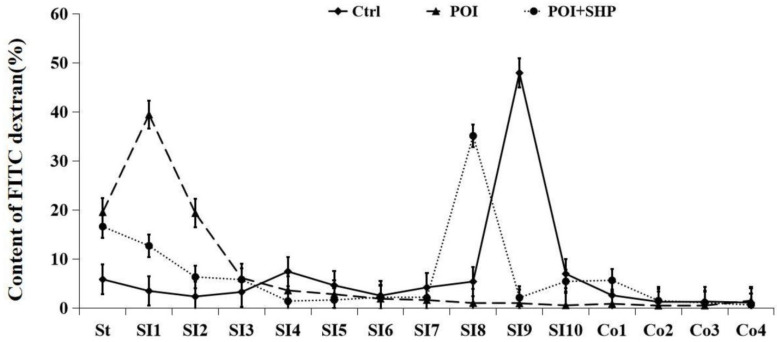
Gastrointestinal transport curves in each group of mice. Note: Gastrointestinal transport curves showing fluorescently labeled dextran in the gastrointestinal tract (St, stomach; SI, small intestine segments 1–10; Co, colon segments 1–4).

Based on the fluorescence labeling values in various parts of the mouse digestive tract, we compared the gastrointestinal motility of the mice. As shown in Figure 1, fluorescently labeled dextran (70 kDa) was rapidly transported in the Ctrl group, with the highest fluorescence value detected in SI9 (the terminal ileum). In the POI group, the highest fluorescence value for dextran was observed in SI1 (the initial ileum), indicating a significant accumulation at the beginning of the gastrointestinal tract, with severe transportation obstruction. In the POI + SHP group, fluorescent dextran accumulated in SI8 (the ileum), showing a significant improvement in gastrointestinal transport compared to the POI group. These results indicate that POI causes significant gastrointestinal motility disorders and a marked decline in gastrointestinal transport function, while the external application of Shenhuang Plaster at the Shenque acupoint can significantly improve these gastrointestinal motility disorders, promoting gastrointestinal peristalsis and enhancing gastrointestinal transport speed.

### 3.3. mRNA Expression of Inflammatory Factors in Small-Intestinal Smooth Muscle of Mice in Each Group

**Figure 2:**
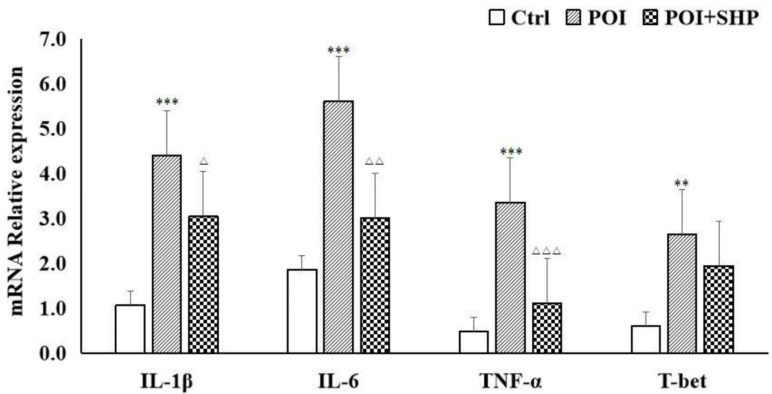
Relative mRNA expression of inflammatory factors in small-intestinal smooth muscle of mice in each group. Note: POI vs. Ctrl, ** *p* < 0.01, *** *p* < 0.001; ^△^ POI + SHP vs. POI, ^△^ *p* < 0.05, ^△△^ *p* < 0.01, ^△△△^ *p* < 0.001.

In this study, we further used qRT-PCR to detect the mRNA expression of the inflammatory mediators IL-1β, IL-6, TNF-α, and T-bet in the smooth muscle tissue of the small intestine. There were significant differences in the expression of inflammatory factors among the groups. Compared with the Ctrl group, the POI group showed significantly increased expression levels of IL-1β, IL-6, TNF-α, and T-bet in the intestinal tissue (Figure 2), indicating a marked inflammatory response in the intestinal tissue of POI model mice. After intervention with SHP, the expression levels of IL-1β, IL-6, and TNF-α mRNA in the intestinal tissue of the POI + SHP group significantly decreased, suggesting that SHP can reduce the mRNA expression of inflammatory mediators in the smooth muscle tissue of the small intestine and alleviate the intestinal inflammation caused by POI.

### 3.4. Changes in Intestinal Microbiota of Mice in Each Group

#### 3.4.1. Quality Control for 16S rDNA Sequencing Data

In this study, a total of 20 samples were sequenced, yielding 1,442,245 raw tags. After data processing and filtering, 1,140,229 clean data sequences were obtained, with an average effective rate of 80%. Compared with the Ctrl group, the average number of features in the POI group significantly decreased. In contrast, the average number of features in the POI + SHP group significantly increased compared to the POI group, approaching the levels seen in the Ctrl group. This indicates that POI reduces the number of features, while the external application of SHP at the Shenque acupoint can increase the number of features. The sequencing information for the samples is shown in Table 5.

#### 3.4.2. Microbial Diversity Analysis

To evaluate the differences in gut microbial diversity among the groups of mice, alpha diversity and beta diversity analyses were conducted. Alpha diversity focuses on the composition and distribution of the microbial community within each sample, while beta diversity examines the differences in microbial community composition and distribution between samples. The results showed significant differences in both alpha and beta diversity in gut microbiota among the groups. The figures and tables in this section are based on Weighted UniFrac analysis.

#### 3.4.3. Alpha Diversity Analysis

**Figure 3:**
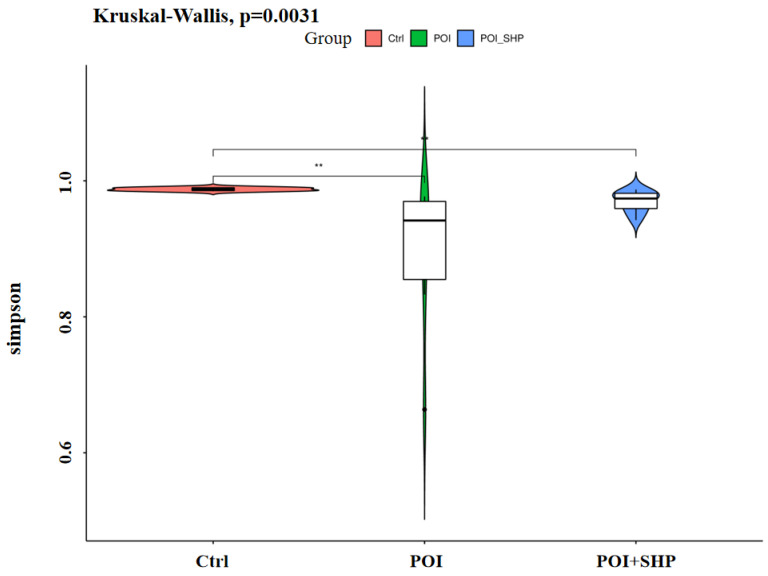
Comparison of the alpha diversity of gut microbiota in each group—Simpson. Note: POI vs. Ctrl, ** *p* < 0.01; POI + SHP vs. POI, ** *p* < 0.01.

The average Good’s Coverage values for all groups were close to 1, indicating sufficient sequencing depth to cover all species within the samples. The observed species and Chao1 indices reflect species richness within the samples (without considering the evenness of each species). As shown in Table 6, compared to the Ctrl group, the mean values for observed species and Chao1 were significantly lower in the POI group (*p* < 0.001). Conversely, the mean values for observed species and Chao1 in the POI + SHP group were significantly higher than those in the POI group (*p* < 0.01). The Simpson index, which reflects both species richness and evenness, was also analyzed. A higher Simpson index indicates greater diversity. Compared to the Ctrl group, the Simpson index was significantly lower in the POI group (*p* < 0.001). Meanwhile, compared to the POI group, it was significantly higher in the POI + SHP group (*p* < 0.01). Rank-sum tests were performed on the Simpson index to identify significant differences in alpha diversity indices among the groups, revealing notable differences (Figure 3). These results suggest that there are significant differences in the alpha diversity of gut microbiota among the groups. The occurrence of POI reduces the alpha diversity of gut microbiota, including species richness and evenness, while the external application of Shenhuang Plaster at the Shenque acupoint significantly increases the species richness and evenness of gut microbiota.

#### 3.4.4. Beta Diversity Analysis

##### PCoA Analysis

**Figure 4:**
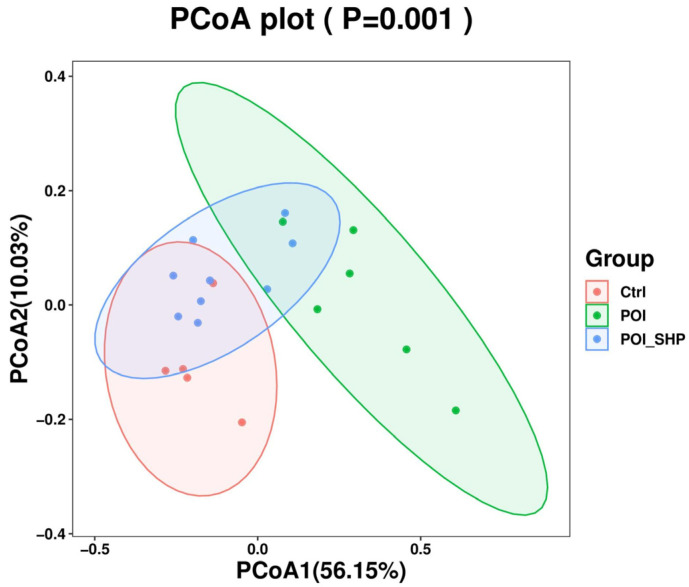
PCoA of gut microbiota in each group of mice.

As shown in Figure 4, there is no clustering between the Ctrl and POI groups; the samples from each group are distinctly distributed, with no overlap. In the POI + SHP group, six samples are clustered closer to the Ctrl group, while three samples are clustered with the POI group. The differences in community composition among the groups are statistically significant (*p* = 0.001).

##### NMDS Analysis

**Figure 5:**
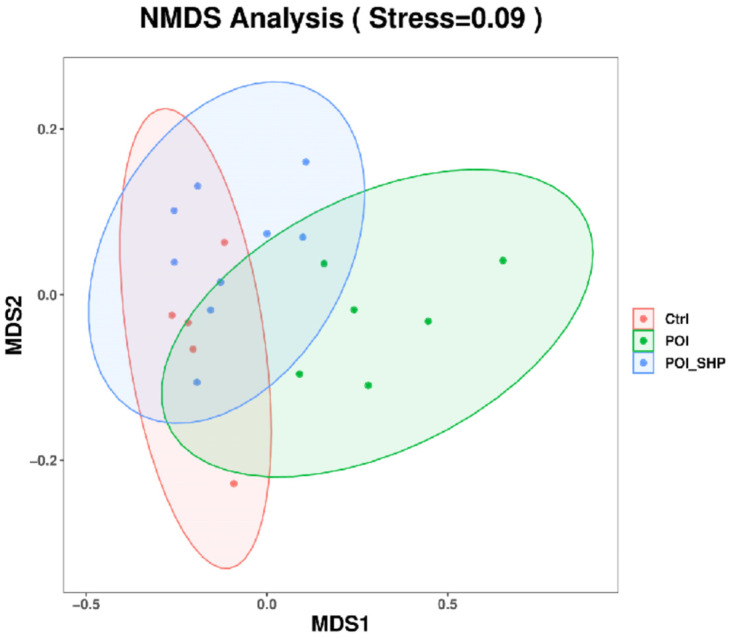
NMDS analysis of gut microbiota in each group of mice.

NMDS analysis, which is unaffected by the absolute values of sample distances and only considers the relative relationships between them, is a nonlinear model that better reflects the nonlinear structure of ecological data. As shown in Figure 5, each point represents a sample, with different-colored points belonging to different groups. The distance between points indicates the degree of difference between samples. The scales on the horizontal and vertical axes represent relative distances. Stress is an indicator of the quality of NMDS analysis results, and a stress value < 0.1 is generally considered a good fit. In this section, NMDS analysis resulted in a stress value of 0.09, which is less than 0.1.

The PCoA and NMDS dimensionality reduction analyses both show a clear separation between the different groups, indicating significant differences among the groups. This suggests that POI model mice exhibit a gut microbiota imbalance, which can be modulated by the external application of SHP at the Shenque acupoint, restoring the gut microbiota composition to normal.

#### 3.4.5. Analysis of Gut Microbiota Structure and Composition

The structure and composition of the gut microbiota in each group of mice are detailed in Table 7 (phylum level) and Table 8 (genus level). At the phylum level, the gut microbiota of the mice primarily consisted of Bacteroidetes, Firmicutes, Proteobacteria, Epsilonbacteraeota, and Deferribacteres. Compared with the Ctrl group, the POI group showed a significant decrease in the relative abundance of Bacteroidetes and Firmicutes, while Proteobacteria abundance was significantly increased. After intervention with Shenhuang Plaster, the relative abundance of Bacteroidetes and Firmicutes in the POI + SHP group increased, with Firmicutes returning to normal levels and a notable decrease in Proteobacteria abundance. At the genus level, the POI group had the highest relative abundance of the pathogenic bacterium Klebsiella in the Enterobacteriaceae family, which significantly decreased after intervention with Shenhuang Plaster in the POI + SHP group. These results indicate that POI model mice exhibit significant microbial dysbiosis, with a substantial enrichment of Gram-negative bacteria playing a crucial role in POI development. The external application of Shenhuang Plaster at the Shenque acupoint can inhibit the growth and proliferation of Gram-negative bacteria and promote the growth of beneficial bacteria.

### 3.5. Serum Metabolomics Analysis of Mice in Each Group

This section discusses the results of high-resolution mass spectrometry for untargeted metabolomics detection in serum samples from mice to further explore the metabolic markers of POI and elucidate the role of gut microbiota and metabolic products in disease development and drug efficacy mechanisms.

#### 3.5.1. Metabolite Quantification—Heatmap Analysis

**Figure 6:**
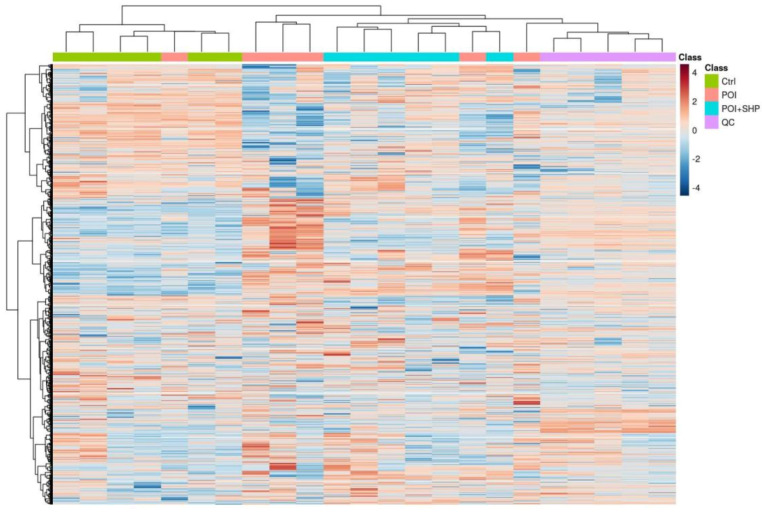
Heatmap of significantly altered metabolites among different groups of mice.

The heatmap illustrates the detection intensity of various metabolites across different samples (intensity values were normalized and scaled). The distance calculation method used was Euclidean, and the clustering method was ward.D2. The heatmap displays the reproducibility and clustering of different metabolites among samples. As shown in Figure 6, there are significant changes in metabolites among the Ctrl, POI, and POI + SHP groups.

#### 3.5.2. Statistical Analysis of Differential Metabolites

##### PLS-DA Analysis

**Figure 7:**
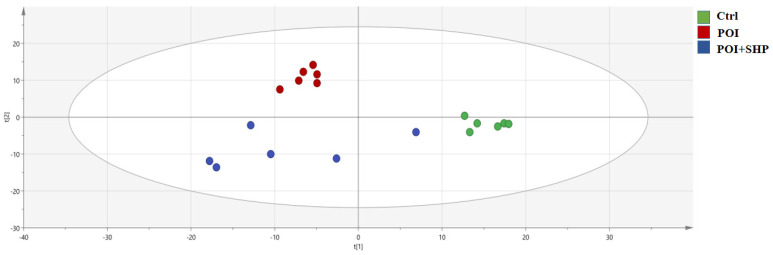
PLS-DA analysis of serum metabolites in each group of mice.

As shown in Figure 7, PLS-DA analysis of metabolites reveals potential differences between the Ctrl group and POI group, as well as between the POI group and POI + SHP group. The distribution of points representing samples in the comparison groups is located across different quadrants, with no overlap between the Ctrl and POI groups. The POI + SHP group tends to cluster closer to the Ctrl group, suggesting that the external application of SHP at the Shenque acupoint may regulate metabolic disorders in POI mice.

##### OPLS-DA Analysis

**Figure 8:**
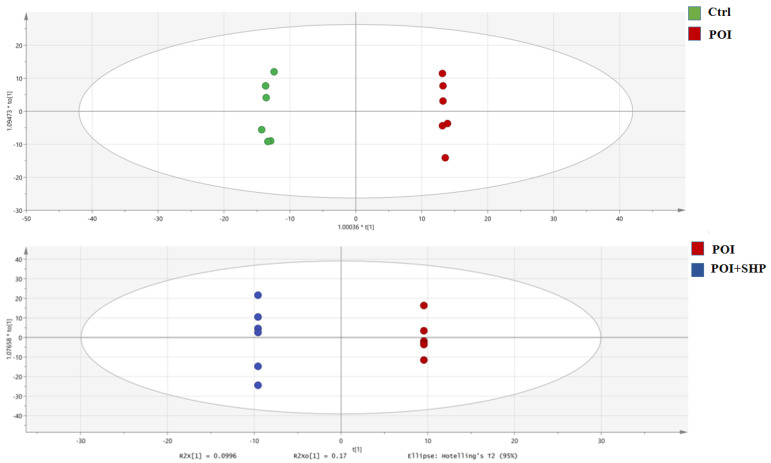
OPLS-DA analysis of serum metabolites in each group of mice.

OPLS-DA, an orthogonal transformation of PLS-DA, removes irrelevant information and improves the model’s interpretability and validity. OPLS-DA was used to identify potential biomarkers associated with POI. As shown in Figure 8, there are significant differences between the Ctrl and POI groups, as well as between the POI and POI + SHP groups. The model validation did not show overfitting (R2Y = 0.99, Q2 = 0.86, both greater than 0.5), indicating that the model accurately describes the samples in each group and can be used for further data analysis.

##### Identification of Differential Metabolites

A total of 48 metabolites were identified (FDR < 0.05, one-way ANOVA for Ctrl vs. POI groups, followed by LSD post hoc test, FC > 1.33 or <0.77, VIP > 1, and correlation coefficient |*r*| > 0.55). The external application of Shenhuang Plaster at the Shenque acupoint significantly regulated 9 out of the 48 serum metabolites in mice (FDR < 0.05, one-way ANOVA for POI vs. POI + SHP groups, followed by LSD post hoc test). These metabolites are L-Carnitine, ε-Caprolactam, Phenylalanine, DL-2-Aminooctanoic acid, Indole-3-pyruvic acid, L-Tyrosine, O-Acylcarnitine, L-Glutamic acid, and Proline (Table 9).

### 3.6. KEGG Pathway Analysis of Differential Metabolites

**Figure 9:**
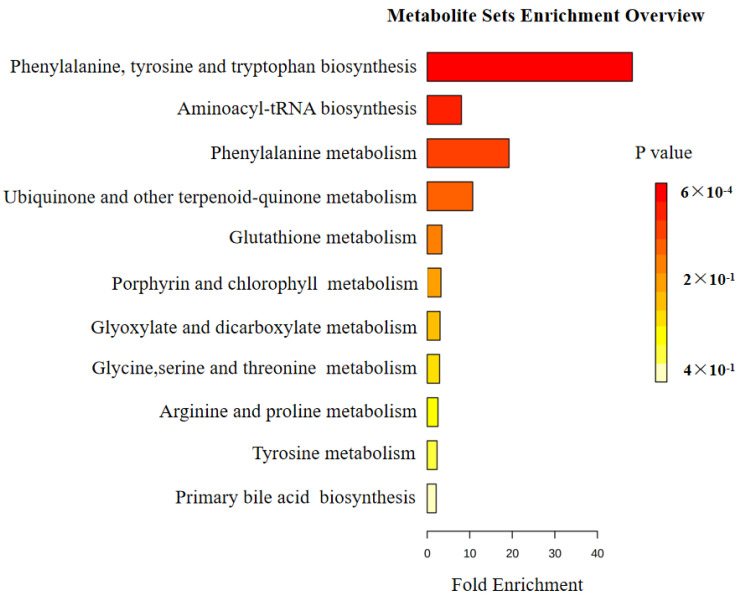
Enrichment pathways of differential metabolites among the three groups of mice.

As shown in Figure 9, 11 metabolic pathways are closely associated with POI intervention using Shenhuang Plaster at the Shenque acupoint. These pathways include the biosynthesis of phenylalanine, tyrosine, and tryptophan; the biosynthesis of aminoacyl-tRNA; the metabolism of phenylalanine; the metabolism of ubiquinone and other terpenoid-quinones; the metabolism of glutathione; the metabolism of porphyrins and chlorophylls; the metabolism of glycolate and dicarboxylates; the metabolism of glycine, serine, and threonine; the metabolism of arginine and proline; the metabolism of tyrosine; and the biosynthesis of primary bile acids. Notably, the pathways for the biosynthesis of phenylalanine, tyrosine, and tryptophan and the metabolism of phenylalanine are the most enriched. These findings suggest that POI may be associated with disturbances in these metabolic pathways and that the application of Shenhuang Plaster at the Shenque acupoint may exert therapeutic effects on POI by modulating these pathways.

### 3.7. Correlation Analysis Between Differential Metabolites and Differential Gut Microbiota

**Figure 10:**
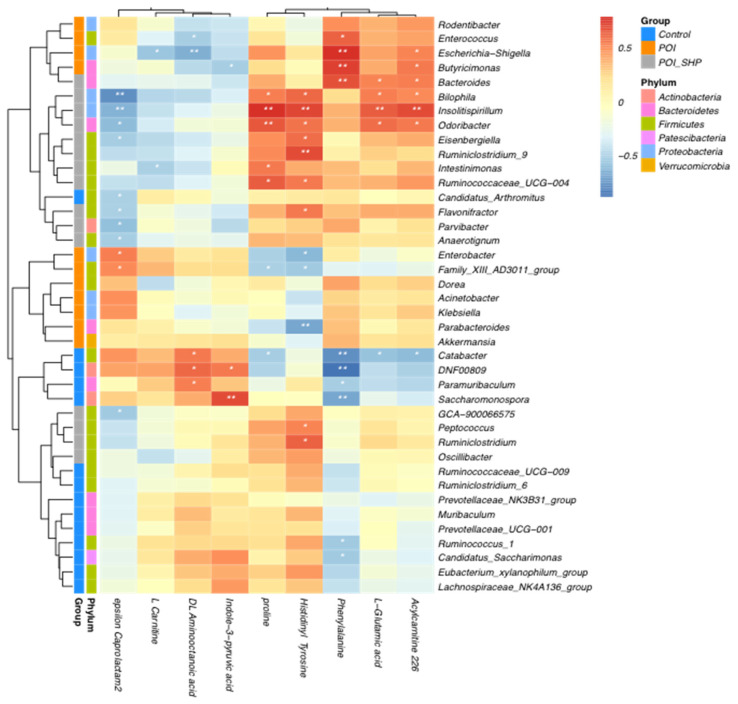
Correlation analysis between differential metabolites and differential gut microbiota. Note: POI vs. Ctrl, POI + SHP vs. POI, * *p* < 0.05, ** *p* < 0.01.

To investigate the impact of the gut microbiota on host metabolic changes, and the regulatory mechanisms behind Shenhuang Plaster targeting the gut microbiota, Spearman correlation analysis was performed between the differential gut microbiota and differential metabolites in the three groups. The results are visualized using a heatmap, shown in Figure 10. In the POI group, pathogenic bacteria such as Enterococcus and Escherichia–Shigella were significantly negatively correlated with the serum concentration of phenylalanine. Escherichia–Shigella was also negatively correlated with the serum concentrations of L-carnitine and DL-2-aminooctanoic acid. Conversely, in the POI + SHP group, the significantly enriched genera Bilophila, Bacteroides, Insolitispirillum, Oscillibacter, Peptococcus, and Ruminiclostridium were significantly positively correlated with the concentrations of proline, histidine, tyrosine, L-glutamic acid, and O-acylcarnitine in the serum. These results indicate that the application of Shenhuang Plaster at the Shenque acupoint may regulate the gut microbiota, modulating amino acid-related metabolic pathways and thereby achieving therapeutic effects in POI.

## 4. Discussion

The recovery of gastrointestinal motility after an operation is affected by many factors, such as the anesthetic drugs, operation method, operation time, and nutritional absorption after surgery. Western medicine usually uses drugs to intervene in POI, such as oral antibiotics, neostigmine, and other cholinesterase inhibitors, which can indeed promote the recovery of gastrointestinal myotonia and have a good effect. However, the use of antibiotics destroys the normal floral structure of the body and reduces the protective function of the intestinal mucosal barrier, and neostigmine leads to bradycardia, bronchospasm, and other adverse reactions [9]. Some studies have also suggested that early postoperative enteral nutrition could significantly promote the recovery of postoperative gastrointestinal motility, but this is not applicable to patients without gastric tubes. Moreover, in these studies, the use of the nutrient solution was strictly controlled clinically; an excessive intake of nutrients would lead to increased abdominal distension and even vomiting and other discomfort [10].

Umbilical application therapy (Shenque point application) is an external treatment method in traditional Chinese medicine, where appropriate dosages of drugs are prepared and applied to the umbilical cord to treat diseases. It is easy to perform, safe, and fast, easily translated to clinical application and promotion, and widely used in internal medicine, surgery, obstetrics and gynecology, pediatrics, and other clinical areas for common diseases. SHP is widely used, particularly externally, in patients during fasting and abstinence after surgery, without any liver and kidney damage or digestive tract irritation.

In this study, we utilized metabolomics to assess the alterations to serum metabolites in POI (postoperative ileus) mice and examined the impact of the application of SHP to the Shenque acupoint on these serum metabolites. We further correlated these findings with gut microbiota data to elucidate the mechanisms by which SHP mitigates POI. KEGG analysis of differential metabolites (Figure 9) indicated significant enrichment in pathways related to phenylalanine, tyrosine, and tryptophan biosynthesis, phenylalanine metabolism, and arginine and proline metabolism. This suggests that the occurrence of POI may be associated with disruptions in the metabolism of these amino acids. External application at the Shenque acupoint may exert therapeutic effects on POI by modulating these metabolic pathways and affecting energy metabolism.

Phenylalanine, an essential amino acid, plays a crucial role in glucose and lipid metabolism [11]. The biosynthetic phenylalanine–tyrosine–tryptophan pathway produces dopamine, norepinephrine, and epinephrine, which are closely linked to glucose and energy metabolism [12]. Ballinger et al. found that the oral administration of 10 g phenylalanine stimulated cholecystokinin release and inhibited energy intake, which was inversely proportional to the dose of phenylalanine [13]. In our study, serum phenylalanine levels were significantly elevated in POI model mice, indicating metabolic abnormalities that may contribute to POI by inhibiting energy intake. Phenylalanine plays a vital role in energy metabolism by delaying gastric emptying and prolonging gastric distention while inhibiting energy intake, which may be one of the main causes of gastrointestinal motility disorders caused by phenylalanine metabolism disorders [14,15,16,17]. Moreover, serum phenylalanine levels in POI model mice positively correlated with the abundance of the pathogenic gut bacteria Enterococcus and Escherichia−Shigella (Figure 10), suggesting a significant positive correlation between gut microbiota dysbiosis and serum metabolite changes in POI. After treatment with SHP, serum phenylalanine levels significantly decreased, indicating that SHP may alleviate the inhibition by phenylalanine of energy intake, regulate energy metabolism, and then alleviate intestinal motility disorders by regulating the metabolic disorders caused by phenylalanine. Additionally, SHP promoted the growth of beneficial gut bacteria and reduced the abundance of pathogenic bacteria, implying that it may regulate phenylalanine metabolism through the modulation of the gut microbiota.

Carnitine, an essential nutrient for transporting fatty acids into mitochondria, is predominantly stored in the muscles. In our study, carnitine levels were significantly reduced in the serum of POI mice, and there was a significant negative correlation between the species abundance of Escherichia−Shigella and serum carnitine levels. Carnitine deficiency can lead to abnormal smooth muscle movement in the gastrointestinal tract, contributing to gastrointestinal discomfort and motility disorders [18]. In the POI + SHP group, carnitine levels were significantly restored, suggesting that SHP application may promote the recovery of gastrointestinal motility in POI mice by increasing serum carnitine levels. Junichiro I et al. demonstrated that carnitine not only improves gastrointestinal function but also modulates the gut microbiota by reducing the abundance of Clostridium species, though the exact mechanisms require further investigation [19]. Simultaneously, SHP can restore carnitine levels, and carnitine itself can improve gastrointestinal function and regulate the gut microbiota. This may create a positive feedback loop, wherein the regulation of the gut microbiota–serum metabolite–gut microbiota axis contributes to therapeutic effects on gastrointestinal motility disorders.

Glutamic acid, an amino acid found in dietary proteins, constitutes 8–10% of the amino acid content in human diets. It is naturally present in protein-rich foods and is also produced by gut microbiota such as Lactobacillus [20,21]. Both prokaryotes and eukaryotes convert glutamic acid to γ-aminobutyric acid (GABA) via glutamate decarboxylase (GAD), and this gene is present in gut bacteria such as Lactobacillus and Bifidobacterium [22]. Glutamic acid serves as a critical energy substrate for the intestinal tissues, contributing to protein metabolism and the synthesis of key molecules (e.g., 2-oxoglutarate, L-alanine, ornithine, arginine, proline, glutathione, and GABA) [23]. About 35% of the energy consumption of the intestinal mucosal cells comes from dietary glutamic acid [24]. The gut is the primary site of dietary glutamic acid absorption and metabolism [25]. The normal gastrointestinal tract has a high capacity to use glutamate, so most glutamate is metabolized in the gut to other amino acids or used as an energy source in the intestinal epithelium [26,27,28]. One result of intestinal glutamate metabolism is that serum glutamate concentration levels are not strongly affected by dietary glutamate, while circulating glutamate levels are maintained at fairly low concentrations [29]. In our study, serum glutamic acid levels were significantly elevated in POI mice, likely due to impaired gut mucosa and enterocytes reducing glutamic acid utilization, affecting its metabolism and energy production and, consequently, gastrointestinal motility. In the POI + SHP group, serum glutamic acid levels significantly decreased, potentially due to SHP protecting intestinal integrity, regulating glutamic acid metabolism, and restoring the gut’s ability to metabolize it, thereby lowering serum levels.

In our study, the Escherichia–Shigella and Klebsiella that show an increased abundance due to POI are potentially pathogenic and pro-inflammatory, resulting in a high expression of cox-2 and iNOS. At the same time, intestinal pathogens can invade intestinal epithelial cells, activate the intracellular signal transduction system, trigger the host immune response, stimulate the secretion of TNF-α, IL-6, and other inflammatory cytokines and mRNA expression, and induce intestinal inflammation [30]. Moreover, the pathogenic bacteria screened were significantly correlated with serum differential metabolites. The literature suggests that small-intestinal smooth muscle inflammation is the core aspect of POI [31]. The results of this study show that SHP could effectively reduce the expression of inflammatory factors. Our previous research results suggested that SHP could effectively inhibit the activation of P-p85, P-AKT, P-IKK, and P-p65, down-regulate the expression of IL-1β, TNF-α, iNOS, and COX-2, and mediate the polarization of macrophages through the PI3K/AKT/NF-κB pathway, thereby improving the inflammatory response in POI [32]. Therefore, we speculate that SHP may exert its effect on POI by inhibiting inflammatory pathways and regulating the intestinal flora and amino acid metabolism. This hypothesis needs to be verified through further multi-omics experiments.

## 5. Conclusions

Clinical studies have found that SHP could effectively promote the recovery of gastrointestinal function in patients with postoperative gastrointestinal insufficiency, promote anal exhaust and defecation, and shorten hospital stays. Applying SHP to the Shenque acupoint in a POI animal model further confirmed that SHP could effectively promote gastrointestinal transmission and reduce the inflammatory response of the small intestine smooth muscle, clarify the effectiveness and safety of SHP in promoting early postoperative gastrointestinal rehabilitation, and enable the creation of a standardized and extendable treatment plan.

Our metabolomics-based study analyzed the regulatory effects of SHP on serum metabolites in POI mice. POI induces gut mucosal damage and enterocyte destruction, leading to decreased carnitine levels and abnormal smooth muscle activity; elevated phenylalanine levels causing metabolic disorders and energy metabolism inhibition, slowing gastric emptying; and a reduced ability to metabolize glutamic acid, resulting in metabolic disruption and elevated serum levels. SHP application to the Shenque acupoint modulated serum phenylalanine, carnitine, and glutamic acid levels, balancing the metabolic disorders induced by POI, restoring amino acid metabolism, and promoting gastrointestinal function recovery. The significant correlation between the abundance of pathogenic gut bacteria (Enterococcus, Escherichia–Shigella) and serum metabolite levels suggests that SHP effectively regulates gut microbiota, reduces pathogenic bacterial abundance, and possibly restores normal amino acid metabolism and gastrointestinal function by modulating gut microbiota and serum metabolites. The results of this study provide an experimental basis for the clinical application of SHP as an intervention for POI. For these achievements, in 2024, we won third prize in the Zhejiang Science and Technology Progress Award and second prize in the Zhejiang Traditional Chinese Medicine Science and Technology Award.

## Figures and Tables

**Table 1:** Experimental reagents.

Reagent Name	Manufacturer
Isoflurane	Lunan Pharmaceutical Co., Ltd., Linyi, China
Polyoxymethylene	Tianjin Institute of Chemistry, Tianjin, China
Anhydrous ethanol	Hangzhou Chemical Reagent Co., Ltd., Hangzhou, China
Trizol	Shenzhen Zike Biotechnology Co., Ltd., Shenzhen, China
Isopropanol	Shenzhen Zike Biotechnology Co., Ltd., Shenzhen, China
DEPC water	Shenzhen Zike Biotechnology Co., Ltd., Shenzhen, China
Fecal DNA Extraction Kit (200) (D4015-02)	OMEGA CORPORATION (Ahmedabad, Gujarat, India)
Fluorescein-labeled dextran	Sigma-Aldrich (St. Louis, MO, USA)

**Table 2:** Experimental apparatus.

Name	Manufacturer
Small Animal Anesthesia Machine	Summit Industry (Chicago, IL, USA)
GNP-9270A water-isolated constant temperature incubator	Ganyi Instruments & Equipment Co., Ltd.
Allegra X-15R Large-capacity Centrifuge	Beckman (Brea, CA, USA)
Ultra-Low Temperature Freezer (DW-HL388)	Hefei Meiling Co., Ltd., Hefei, China
Super Clean Bench	Samsung Electronics Co., Ltd. (Suwon-si, Republic of Korea)
Autoclave	SANYO (Osaka, Osaka, Japan)
AG204 Analytical Balance	Mettler Toledo (Columbus, OH, USA)
LightCycler PCP	Roche (Basel, Switzerland)
Centrifuge 5417R	Eppendorf (Hamburg, Germany)
Eddy current oscillator (WH-861 eddy current oscillator)	HuaLiDaf
NovaSeq Sequencing System	Illumina, courtesy of Leobiot, San Diego, CA, USA
SYNAPT G2-Si QTOF/MS	Waters (Milford, MA, USA)
Sample Manager and Acquittieu Pooler Quaternary Pump	Waters
UPLC-TripleTOF5600Plus	Sissy, courtesy of Leobiot

**Table 3:** qRT-PCR primer sequence list.

Factor	Forward Primer (5′ to 3′)	Reverse Primer (5′ to 3′)
β-actin	TTCCAGCGTTCCTTCTTGGGT	GTTGGCATAGAGGTGTTTACG
IL-1β	TCATGGGATGATGATAACCTGCT	CCCATACTTTAGGAAGACACGGATT
IL-6	CTTTTGAIATATGGAAT	CCAGTTTGGTAGGCATCCATC
T-bet	CAAGTGGGTGCAGTGTGGAAAG	TGGAGAGACTGCAGGACGATC
TNF-α	CCCTCACACTCAGATCATCTTC	GTTGGTTGTCTTTGAGATCCAT

**Table 4:** Changes in body weight of mice in each group.

Group	Initial Weight (g)	Final Weight (g)	Weight Difference (g)
Ctrl	21.65 ± 1.47	23.05 ± 1.62	2.23 ± 0.94
POI	20.46 ± 0.83	18.80 ± 0.82	−1.84 ± 0.66 ***
POI + SHP	20.96 ± 2.25	20.88 ± 2.45	−1.00 ± 0.10 ^△△△^

Note: POI vs. Ctrl, *** *p* < 0.001; POI + SHP vs. POI, ^△△△^ *p* < 0.001.

**Table 5:** Sample sequencing information summary table.

Sample	Group	Raw_Tags	Raw_Bases	Valid_Tags	Q30%	GC%
Ctrl-1	Ctrl	76,915	32.01 M	76,915	93.79	54.69
Ctrl-2	Ctrl	73,975	30.89 M	73,975	91.87	53.72
Ctrl-3	Ctrl	75,671	31.36 M	75,671	94.13	54.15
Ctrl-4	Ctrl	70,523	29.20 M	70,523	90.01	53.95
Ctrl-5	Ctrl	70,971	29.39 M	70,971	89.22	54.54
POI-1	POI	68,969	29.29 M	68,969	92.99	54.10
POI-2	POI	72,957	30.75 M	72,957	92.60	53.82
POI-3	POI	74,306	31.16 M	74,306	93.44	53.75
POI-4	POI	68,054	28.54 M	68,054	93.48	52.65
POI-5	POI	67,314	28.07 M	67,314	91.14	52.53
POI-6	POI	74,575	31.34 M	74,575	93.55	51.49
POI + SHP-1	POI + SHP	76,730	31.78 M	76,730	90.73	53.16
POI + SHP-2	POI + SHP	64,761	27.02 M	64,761	92.36	52.85
POI + SHP-3	POI + SHP	77,266	31.91 M	77,266	93.33	53.67
POI + SHP-4	POI + SHP	67,721	28.29 M	67,721	92.81	52.25
POI + SHP-5	POI + SHP	71,560	29.53 M	71,560	93.28	54.40
POI + SHP-6	POI + SHP	70,563	29.30 M	70,563	91.06	53.78
POI + SHP-7	POI + SHP	73,471	30.51 M	73,471	93.48	53.13
POI + SHP-8	POI + SHP	72,024	29.78 M	72,024	93.63	54.09
POI + SHP-9	POI + SHP	73,919	30.66 M	73,919	91.37	53.71

**Table 6:** Comparison of the alpha diversity of gut microbiota in each group of mice.

Group	Observed Species	Chao1	Simpson
Ctrl	994.40 ± 197.93	999.79 ± 202.88	0.99 ± 0.01
POI	505.00 ± 131.92 ***	509.44 ± 133.27 ***	0.89 ± 0.12 ***
POI + SHP	803.11 ± 166.71 ^△△^	807.13 ± 169.44 ^△△^	0.97 ± 0.01 ^△△^

Note: POI vs. Ctrl, *** *p* < 0.001; POI + SHP vs. POI, ^△△^ *p* < 0.01.

**Table 7:** Phylum-level gut microbiota structure and composition in each group of mice (%).

Phylum	Ctrl	POI	POI + SHP
Bacteroidetes	52.22	39.95 ↓	44.08 ↑
Firmicutes	37.10	15.61 ↓	37.46 ↑
Proteobacteria	4.39	35.97 ↑	11.67 ↓
Epsilonbacteraeota	1.95	2.51	2.97
Verrucomicrobia	0.09	3.01	1.59
Deferribacteres	1.38	1.37	0.29
Actinobacteria	0.44	1.22	0.60
Patescibacteria	1.57	0.12	0.70

Note: POI vs. Ctrl, ↓ means decreased; POI + SHP vs. POI, ↑ means rised.

**Table 8:** Genus-level gut microbiota structure and composition in each group of mice (%).

Genus	Ctrl	POI	POI + SHP
Muribaculaceae_unclassified	38.21	12.95	17.94
Bacteroides	2.49	8.56	10.05
Escherichia-Shigella	0.02	9.78	6.39
Klebsiella	0.01	17.35	0.05
Eisenbergiella	4.34	0.82	7.41
Lachnospiraceae_NK4A136_group	6.25	0.50	3.41
Rikenellaceae_RC9_gut_group	1.33	2.76	4.16
Parabacteroides	1.14	6.21	1.76
Firmicutes_unclassified	4.23	0.47	3.53

**Table 9:** Potential biomarkers and metabolic pathways involved in POI intervention through the external application of SHP at the Shenque acupoint.

No.	Metabolite	Molecular Formula	Molecular Weight (*m*/*z*)	Retention Time (min)	Regulation		Associated Pathways
					POI *	POI + SHP ^△^	
1	L-Carnitine	C_7_H_15_NO_3_	162.11	0.85	↓	↑	Lipid metabolism
2	Phenylalanine	C_9_H_11_NO_2_	166.09	2.21	↑	↓	Phenylalanine metabolism
3	Histidinyl Tyrosine	C_9_H_11_NO_3_	182.08	1.42	↑	↓	Phenylalanine metabolism
4	O-Acylcarnitine	C_8_H_14_NO_4_R	472.34	6.18	↑	↓	Lipid metabolism
5	ε-Caprolactam	C_6_H11NO	114.07	8.56	↓	↑	Microbial metabolism in diverse environments
6	DL-2-Aminooctanoic acid	C_8_H_17_NO_2_	160.13	3.75	↓	↑	Protein/amino acid biosynthesis
7	L-Glutamic acid	C_5_H_9_NO_4_	130.05	5.08	↑	↓	Glutathione metabolism
8	Indole-3-pyruvic acid	C_11_H_9_NO_3_	221.09	1.81	↓	↑	Phenylalanine, tyrosine, and tryptophan biosynthesis
9	Proline	C_5_H_9_NO_2_	144.10	0.87	↑	↓	Aminoacyl-tRNA biosynthesis

Note: POI vs. Ctrl, * *p* < 0.05; POI + SHP vs. POI, ^△^ *p* < 0.05; ↓ means decreased; ↑ means rised.

## Data Availability

Data will be made available upon request due to the policies and confidentiality agreements adhered to in our laboratory.
